# Can post stroke walking improve via telerehabilitation? A systematic review in adults with stroke

**DOI:** 10.3389/fresc.2023.1154686

**Published:** 2023-05-17

**Authors:** Shravni Deshmukh, Sangeetha Madhavan

**Affiliations:** ^1^ Department of Physical Therapy, Brain Plasticity Laboratory, College of Applied Health Sciences, University of Illinois at Chicago, Chicago, IL, United States; ^2^ Graduate Program in Rehabilitation Science, College of Applied Health Sciences, University of Illinois at Chicago, Chicago, IL, United States

**Keywords:** stroke, telerehabilitation, systematic review, walking, gait

## Abstract

**Objectives:**

The purpose of this systematic review is to analyze primary studies investigating the effects of telerehabilitation on walking outcomes for the treatment of adult stroke survivors.

**Methods:**

Data sources included PubMed, Embase and CINAHL searched until August 2022, using combinations of several keywords such as “telerehabilitation”, “stroke”, and “gait”. Studies were required to have bidirectional form of videoconferencing with assessor presence, and include assessment of walking function (speed, endurance and/or balance). Data extraction was performed from each full text by one author, and quality and bias were assessed using the Physiotherapy Evidence Database (PEDro).

**Results:**

Eight studies involving 248 participants met the inclusion criteria. Seven reported significant improvements in outcomes of balance and two showed improvements in endurance after telerehabilitation. Two studies observed greater balance improvements in the telerehabilitation group compared to control and/or in-person therapy. Differences in frequency, training duration, intervention type, and absence of an in-person therapy control group were identified as causes of variation between studies.

**Conclusions:**

The effectiveness of telerehabilitation as a mode of therapy for walking could not be definitively determined due to the limited number of studies that directly measured walking speed or endurance. However, strong evidence was found for the use of telerehabilitation for balance improvements, which has implications for walking recovery.

**Impact statement:**

Telerehabilitation appears to be safe, feasible and demonstrated high adherence. Our results highlighted limited studies using real-time supervision to administer telerehabilitation and lack of studies focusing on outcomes of walking speed and endurance, needed to fully determine the role of telerehabilitation for gait recovery.

**Systematic review registration number:**

PROSPERO number CRD42021238197.

## Introduction

Stroke is the leading cause of adult-onset disability resulting in permanent motor impairment, decrease in activities of daily living including community ambulation, and reduced quality of life (1–3). Stroke survivors benefit from intense therapy that is provided during acute and subacute phases of stroke; however, the intensity and dosage of therapy declines after the initial treatment period. Although rehabilitation persists to be effective even decades post stroke, stroke survivors are unable to receive the necessary dosage of supervised functional practice due to various factors including health care costs, insurance coverage for long periods of therapy, transportation to and from the clinic, and provider shortages (4, 5).

Telerehabilitation, a branch of telemedicine, is an alternative way to deliver conventional rehabilitation services to patients in a remote location using telecommunication technologies (6). Telerehabilitation is a rapidly increasing field as delivery of therapy to patients in their own home enables broader access to healthcare, reduced travel time for patients to visit the clinic or clinicians to visit patient homes, and possibly increased doses of therapy (7). Studies examining upper limb stroke functional training using telerehabilitation have demonstrated that treatment delivered via telerehabilitation is similar to in-person treatment in terms of motor recovery, health-related quality of life, caregiver strain and patients' satisfaction (8–10). Some studies also reported that telerehabilitation interventions resulted in greater improvements in health-related quality of life, decrease in caregiver strain, and increase in patient satisfaction compared to conventional face-to-face therapy (9–13). While telerehabilitation appears to be a promising approach to increase access to supervised upper limb therapy, effective interventions to improve walking after stroke, typically characterized by intensive and repeated stepping movements in standing performed overground or on the treadmill, create concern for participant safety (loss of balance and falls) when delivered via telerehabilitation. Few walking and balance studies have successfully implemented gait related interventions for stroke via telerehabilitation. However, results have been conflicted with reports demonstrating superiority of telerehabilitation compared to conventional therapy or no difference between the two. While systematic reviews on telerehabilitation have been conducted previously, these studies have combined results from upper and lower limb interventions, making results inconclusive for walking (9, 10, 14). A recent scoping review by Ramage et al. focused on telehealth interventions in weight-bearing activities or standing positions to primarily highlight safety, efficacy, and feasibility of lower limb telerehabilitation without addressing measures of walking ability (15). Thus, the evidence for the effectiveness of telerehabilitation to improve walking function in stroke remains unclear. To fill this gap in knowledge, this systematic review aims to examine the current state of telerehabilitation for improving walking outcomes in adults with stroke. To improve the rigor of this review, we selected and analyzed studies that included remotely supervised two-way delivery as the mode of training.

## Methods

Our study protocol was developed in accordance with PRISMA (Preferred Reporting Items for Systematic Review) guidelines and was registered with the International Prospective Register of Systematic Reviews (PROSPERO, number CRD42021238197).

### Data sources and searches

A systematic literature search through August 2022 was carried out in the following electronic databases: PubMed, EMBASE and CINAHL. A combination of the following search terms was used with Boolean operators “AND” and “OR” for a thorough search:

(“telerehabilitation” OR “tele-rehabilitation” OR “e-rehabilitation” OR “videoconferencing” OR “telemedicine” OR “telehealth” OR “erehabilitation” OR “tele rehabilitation” OR “video-conferencing” OR “mHealth” OR “eHealth” OR “Mobile Health” OR “remote supervision” OR “tele supervision” OR “teleconsult” OR “telecommunication” OR “telecare” OR “teletherapy” OR “tele therapy” OR “remote consult” OR “remote consultation” OR “tele-rehabilitation” OR “tele-coaching” OR “tele homecare” OR “tele conference”) AND (“stroke survivors” OR “Acute Cerebrovascular Accidents” OR “Acute Cerebrovascular Accident” OR “Apoplexy” OR “Brain Vascular Accident” OR “Brain Vascular Accidents” OR “Cerebrovascular Accident” OR “Cerebrovascular Accidents” OR “Cerebrovascular Apoplexy” OR “Cerebrovascular disease” OR “CVA” OR “CVAs” OR “stroke” OR “strokes” OR “Brain Ischemia” OR “Hemiplegia” OR “Paresis”) AND (“walking function” OR “gait” OR “balance” OR “walking” OR “walk” OR “endurance” OR “Ambulation” OR “lower limb” OR “step count” OR “stride” OR “walks”)

### Study selection

**Figure 1:**
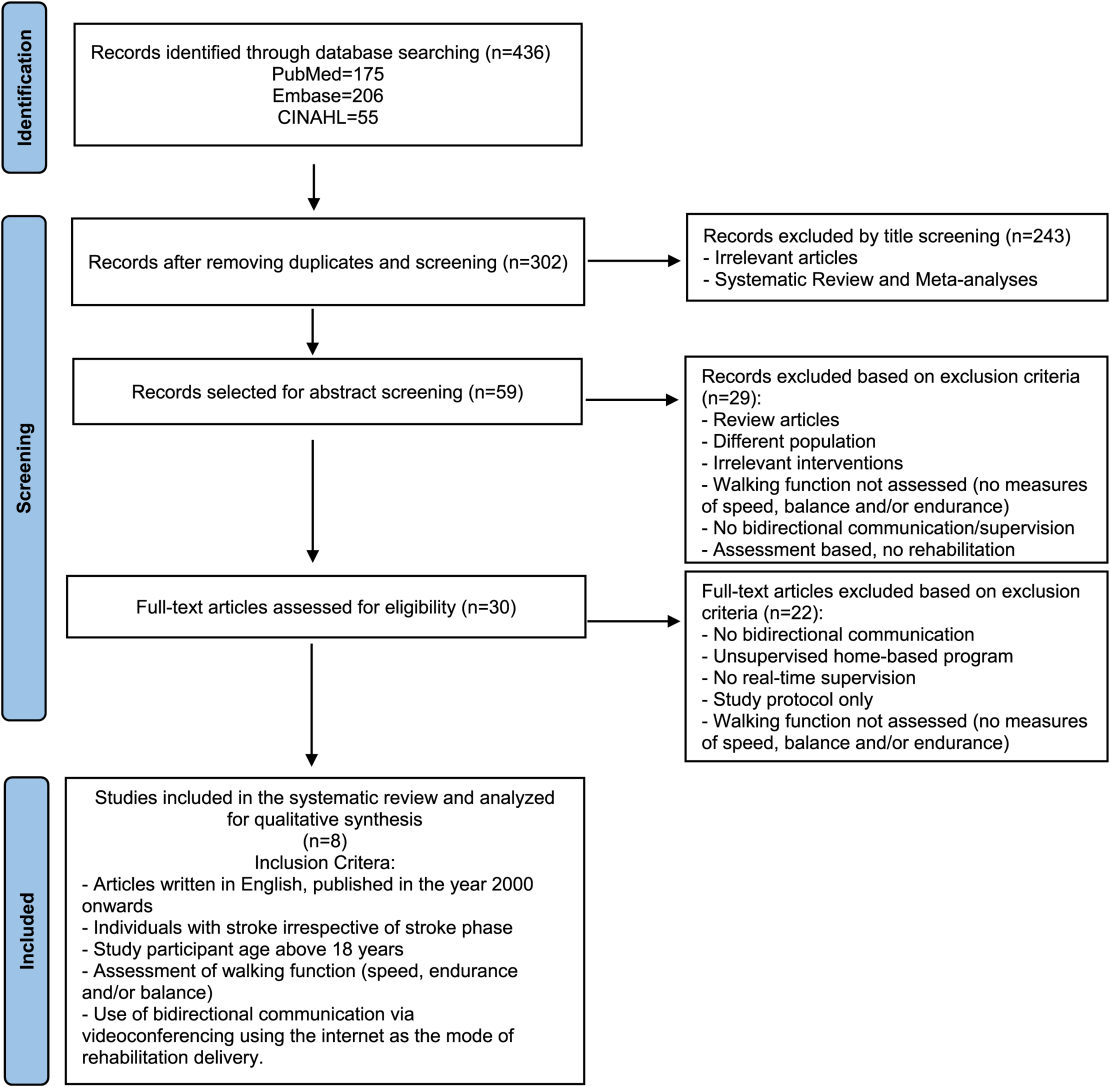
Article selection process. Flowchart displaying study selection process using PubMed, Embase and CINAHL databases. The number of searches obtained in the database search as well as the number of studies remaining after the inclusion and exclusion criteria were applied are shown.

The initial search revealed 436 citations. After removal of duplicate articles, study titles, abstracts and eventually full-text studies were screened based on the following inclusion criteria: timeline restricted to the year 2000 onwards, articles written in English, individuals with stroke irrespective of stroke phase, study participant age above 18 years, assessment of walking function (speed, endurance and/or balance), use of bidirectional communication via videoconferencing using the internet as the mode of rehabilitation delivery and publication type as human clinical studies. Studies were excluded if they were categorized as reviews and meta-analyses and included only telephonic consultations or home-based exercises without video conferencing and/or remote supervision by research personnel. Through this article selection process, highlighted in Figure 1, eight citations were narrowed and agreed upon by both authors.

### Data extraction and quality assessment

Eligible full text extraction was carried out for all included studies by both authors using the predetermind eligibility criteria. All eight studies were assessed and appraised for quality using the PEDro scale. The PEDro scale is widely used to assess and measure the methodological quality of randomized controlled trials. It consists of a total of 11 items, out of which the first item does not count towards the final score (16, 17). Depending on the PEDro score for each study, ranging from 0 to 10, each study was categorized as poor (<4), fair (4–5), good (6–8) or excellent (9–10) quality (18). PEDro scores were determined by one author, however when confirmed scores were available on the PEDro website, the author checked for agreement between self-rated and confirmed scores.

### Data synthesis and analysis

The authors conducted a narrative synthesis of the included studies. Target findings of each study were identified and synthesized. Study characteristics (author, publication year and sample size) (Table 1), participant characteristics (sex, age, phase of stroke, stroke type and side of lesion) (Table 1), characteristics of the control and intervention group (type of intervention, number of sessions, frequency, training, and session duration) (Table 2) and results (Table 3) were summarized for each article. Similarities and differences of descriptive characteristics between studies were examined.

**Table 1:** Study participant characteristics.

Study	Total participants *n* = 248	Participants in the intervention group *n* = 147	Sex (M/F)	Age (years) mean ± SD	Phase of stroke	Stroke type (ischemic/hemorrhagic)	Lesion side (L/R)
Bernocchi et al. (19)	23	23 (No controls)	16/10	70 ± 10	Subacute stroke: *n* = 13; Chronic stroke: *n* = 10	23/3	14/12
Chen et al. (20)	54	27	33/21	C: 66 ± 12 I: 67 ± 12	Acute stroke	46/8	—
Chen et al. (21)	30	15	18/12	median (IQR) C: 61 (53–68) I: 60 (52–68)	Chronic stroke	17/13	16/14
Deng et al. (22)	16	16	11/5	median (IQR) 55 (51–62)	Chronic stroke	—	5/11
Huzmeli et al. (23)	10	10 (No controls)	6/4	53 ± 7	Chronic stroke	—	5/5
Lin et al. (24)	24	11	17/7	C: 76 (3) I: 75 (2)	Chronic stroke	—	11/13
Lloréns et al. (25)	30	15	17/13	C: 56 ± 7 I: 56 ± 10	Chronic stroke	19/11	18/12
Wu et al. (26)	61	30	36/25	C: 59 ± 9 I: 57 ± 12	Acute stroke	44/17	38/23

C, control group; I, intervention group receiving telerehabilitation; IQR, interquartile range (Q1–Q3).

**Table 2:** Training protocol characteristics.

Study	Control	Telerehabilitation	Number of sessions	Frequency (sessions/week)	Training duration (weeks)	Session duration (minutes)
Bernocchi et al. (19)	No control	Physical therapy exercises consisting of postural changes, trunk movements, sit to stand, upper limb exercises, walking, stair climbing, and activities of daily living, nurse tutor advice and drug therapy changes.	Not provided	2 (54% patients) 3 (31% patients)	12	Not provided
Chen et al. (20)	Same as intervention but conducted in person	Electromyography-triggered neuromuscular stimulation (ETNS) for 20 min, upper and lower limb physical therapy exercises, balance and walking training and occupational therapy.	60	Twice per working day	12	60
Chen et al. (21)	Conventional physical therapy exercises (warm up, sit to stand transitions, balance exercises, standing, walking, neuromuscular facilitation, strengthening and cool down)	Virtual reality program—target-oriented stepping task, multidirectional reaching task, Tai Chi exercises for balance and posture	12	3	4	40
Deng et al. (22)	No control, both groups received different complexities of the same movement protocol	Simple (move group) and complex ankle movement training (track group) via a training system shown of the laptop screen	20	Participants selected their own daily schedule but maximum of 60 training blocks per day	3 (20 days)	Not provided
Huzmeli et al. (23)	No control	Neurodevelopmental theory-based treatment protocol consisting of upper limb, lower limb, and trunk exercises	9	3	3	Not provided
Lin et al. (24)	Conventional balance training	Balance training conducted via a 3D interface including static and dynamic training.	12	3	4	50
Lloréns et al. (25)	Same as intervention but conducted in person	Balance training via a virtual reality program (Microsoft Kinect) including stepping exercises, weight shifts and dynamic postural adaptation.	20	3	7	45
Wu et al. (26)	Rehabilitation guidance via telephone.	Positioning, early mobility, balance, and gait training during the acute phase along with strengthening and task specific activities of daily living (ADLs) training during the recovery phase.	24	2	12	Not provided

**Table 3:** Outcome measures and significant results.

Study	Time of assessment	Outcome measures
Bernocchi et al. (19)	Pre and post	Tinetti Perfomance-Oriented Mobility Assessment* Berg Balance Scale* Nine-hole Peg Test* Motricity Index* 6 Minute Walk Test* Modified Barthel Index* Beck's Depression Inventory* Family Strain Questionnaire* Satisfaction Questionnaire*
Chen et al. (20)	Pre, mid and post	Modified Barthel Index* Berg Balance Scale* Caregiver Strain Index* Modified Rankin Scale* Electromyography*
Chen et al. (21)	Pre and post	Timed Up & Go Test^#^ Berg Balance Scale* Motricity Index Functional Ambulation Category Modified Falls Efficacy Scale
Deng et al. (22)	Pre and post	Quantitative Gait Kinematic Parameters – Gait temporal symmetry ratio* – Ankle Dorsiflexion* and Plantarflexion range – Toe clearance – Stride length Accuracy Index* 10-meter Walk Test Functional Magnetic Resonance Imaging
Huzmeli et al. (23)	Pre and post	Berg Balance Scale* Mini-Mental State Examination 36-Item Short Form Survey
Lin et al. (24)	Pre and post	Berg Balance Scale* Barthel Index* Satisfaction Questionnaire
Lloréns et al. (25)	Pre, post and 1 month follow up	Berg Balance Scale* Brunel Balance Assessment* Performance-Oriented Mobility Assessment balance subscale* Performance-Oriented Mobility Assessment gait subscale* System Usability Scale (only post) Intrinsic Motivation Inventory (only post)
Wu et al. (26)	Pre, 2 mid and post	Berg Balance Scale^#^,^*^ Timed Up & Go Test^#^,^*^ Modified Barthel Index^#^,^*^ Stroke Specific Quality of Life^#^,^*^ 6 Minute Walk Test* Fugl Meyer Assessment*

* Statistically significant improvement in both groups.

^#^ Greater statistically significant improvement in the telerehabilitation group.

### Role of the funding source

The authors did not receive funding for the submitted work and have no conflicts to report.

## Results

### Study characteristics

**Figure 2:**
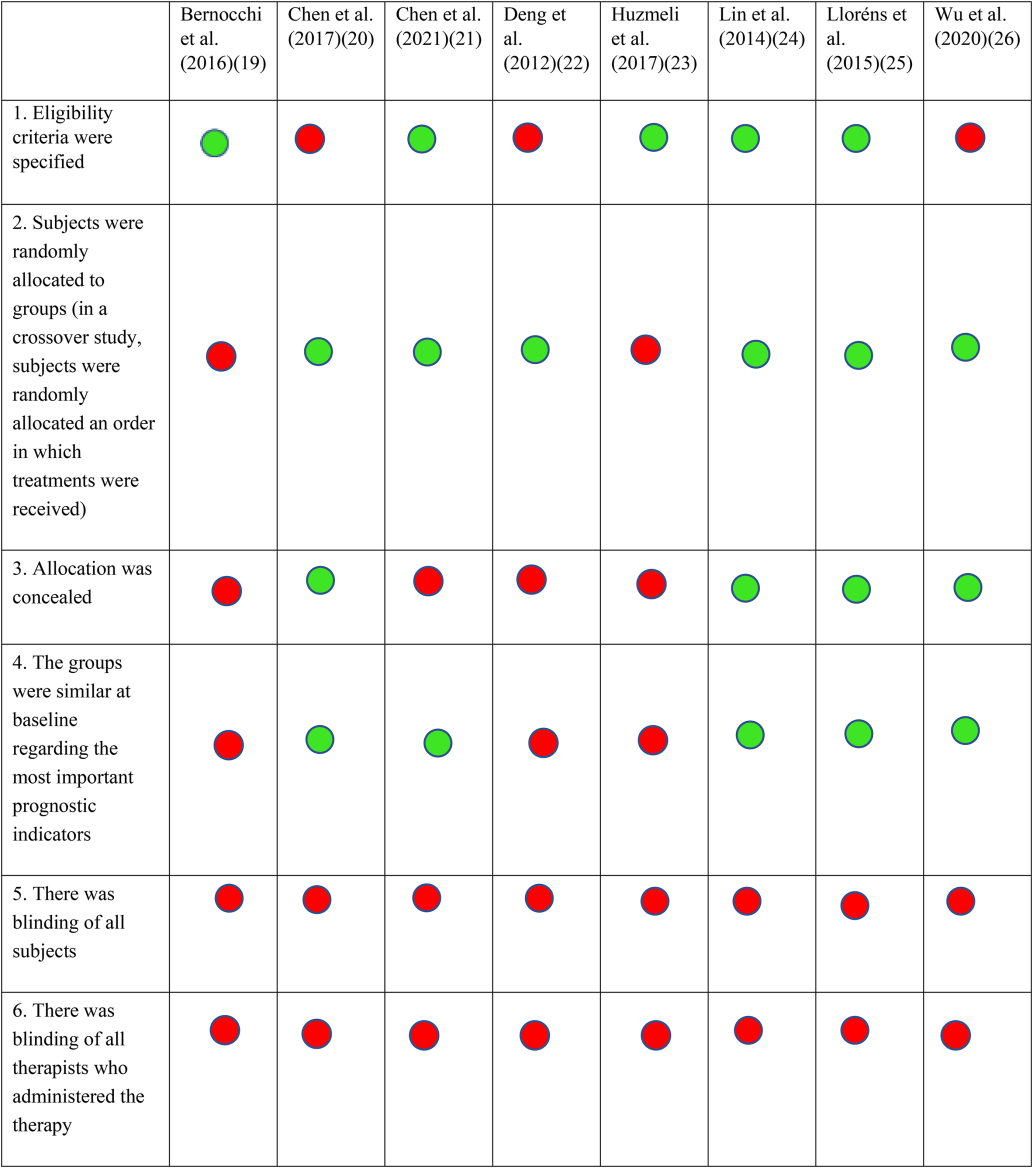
PEDro scores. Each included study was scored using the Physiotherapy evidence database (PEDro) tool's criteria. A green circle indicates that the specific criteria was met, and a red circle shows that the criteria was not met. The total PEDro score is shown at the bottom of each column.

Eight studies were found to satisfy the predefined inclusion criteria (Figure 1) (19–26). The remaining studies were mostly excluded because the mode of delivery did not involve bilateral communication or real-time supervision, or the protocol involved a research personnel at the patient's home for the duration of the intervention. Other studies that were excluded did not assess the effects of telerehabilitation on walking function or were only in the protocol stage. Half of the included studies were rated as good, and the other half were rated fair, based on their PEDro scores (Figure 2). All studies consistently reported between-group differences in at least one key outcome measure. Due to the nature of the interventions, blinding the participant or the therapist was not possible in all studies.

**Figure:**
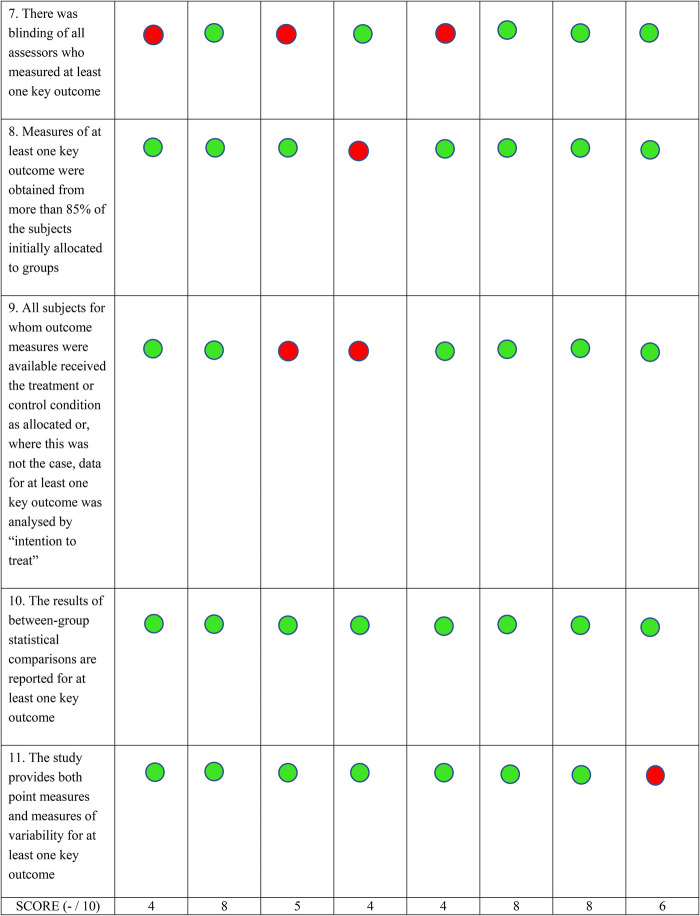


### Participant characteristics

Participants were between the ages of 50–80 years of age with a higher number of males. Three studies recruited patients in the acute phase (20, 23, 26), whereas four studies recruited chronic phase patients (21, 22, 24, 25), and one study included a mix of subacute and chronic participants (19). The sample size of stroke participants in the telerehabilitation group was 147, while the overall total was 248 including participants in the control arm (Table 1).

### Details of intervention

Interventions administered in the acute phase comprised of electromyography-triggered neuromuscular stimulation (ETNS) paired with exercises (20) and conventional physical therapy (PT) exercises (26). The study by Huzmeli et al. which recruited both sub-acute and chronic phase participants performed conventional physical therapy (PT) exercises (23). Studies administered in the chronic phase included a virtual reality program for balance and posture (21, 24, 25) and an ankle movement program (22). Three studies (19, 22, 23) did not have a control group and one study (22) used 2-way video conferencing in both the control as well as intervention group. Interventions administered in control groups included conventional physical therapy, in-person delivery of the same protocol used via telerehabilitation or rehabilitation guidance via telephone. The duration of treatment observed across studies spanned 3–12 weeks (approximately 9–60 sessions). Means of implementation of telerehabilitation was using either standard videoconferencing platforms (e.g., Skype), custom designed platforms or virtual reality (Table 2).

### Effects of intervention

To estimate improvements in walking function, we focused on statistically significant differences (*p* ≤ 0.05) in measures of walking speed, walking endurance, and balance. The 10-meter walk test (10MWT) was used to measure walking speed. Walking endurance was examined using the Six-minute walk test (6MWT). Most studies measured balance using the Berg balance scale (BBS), Brunel balance assessment (BBA), Timed up & go test (TUG) or the Tinetti Performance Oriented Mobility Assessment (POMA).

### Walking outcomes

Deng et al. performed the 10-meter walk test (10MWT) to measure gait speed and did not see statistically significant improvements in this measure (22). The Six-minute walk test (6MWT) which measures ambulatory capacity and walking endurance was reported by two studies (19, 26). A statistically significant increase in the distance covered was seen after training in both groups with no between-group differences. Lloréns et al. administered the Tinetti Performance Oriented Mobility Assessment gait scale, which measures walking ability and a statistically significant improvement in scores was seen in the control and telerehabilitation group (25).

Seven (19–21, 23–26) of the eight studies used the BBS as an outcome measure for assessing dynamic postural balance and reported a statistically significant increase in balance for both the control and telerehabilitation groups. Wu et al. showed a greater statistically significant improvement in balance in the telerehabilitation group when compared to the control group (26). Chen et al. (21) and Wu et al. (26) additionally used the TUG test which measures balance, fall risk and functional mobility. A statistically significant difference was seen between the control and intervention groups, with the telerehabilitation group showing a greater decrease in time required to perform the task. Another outcome used by Bernocchi et al. (19) and Lloréns et al. (25) was the POMA scale which measures gait and balance status, and an improvement in scores was seen in both the control and telerehabilitation group. Lloréns et al. reported statistically significant improvements on the BBA, which assesses functional balance (25). Chen et al. also administered the Modified Falls Efficacy Scale (MFES) that measures perceived fall risk, and a statistically significant improvement was seen after both interventions (21). Chen et al. observed no significant within or between group differences on the Functional Ambulation Category (FAC) which evaluates walking ability depending on how much support is needed for balance during walking (21).

### Activity, participation, and quality of life

Wu et al. used the Stroke Specific Quality of Life Questionnaire (SS-QOL) to assess changes in perception of quality of life and reported a significantly greater improvement in several domains (family role, language, activity ability, self-care ability, social role, and upper extremity function) in those who participated in telerehabilitation compared to controls (26). Huzmeli et al. used the SF-36 quality of life questionnaire but did not report a significant change in the scores after telerehabilitation (23). Three studies assessed functional independence using the Modified Barthel Index (19, 20, 26). All of them (19, 20, 26) reported significant improvements, out of which one (26) reported a significant increase in the scores with telerehabilitation compared to control. Lin et al. used the Barthel Index and reported significant increase in scores indicating an improvement in functional independence in both the control and telerehabilitation group (24). Chen et al. reported significant improvements in the scores for the Modified Rankin Scale (MRS), indicating reduced disability levels after both the interventions, with no significant between group differences (20).

### Caregiver related outcomes

Chen et al. used the Caregiver Strain Index (CSI) to assess caregiver burden and reported a significant improvement in the scores for both groups, suggesting that caregiver burden decreased, but no between group differences were observed (20). Bernocchi et al. administered the Family Strain Questionnaire (FSQ-SF) which assesses challenges faced by the caregiver, and an improvement in scores was seen with telerehabilitation, indicating a reduction in family strain (19).

### Other outcomes

Wu et al. used the Fugl Meyer Assessment scale (FMA) for assessing upper extremity, lower extremity, and total performance-based motor impairment (26). They reported an increase in the FMA scores in both groups with no between group differences, indicating a reduction in level of motor impairment. Telerehabilitations groups in studies done by Chen et al. (21) and Bernocchi et al. (19) reported a significant increase in the Motricity index (MI), which also assesses motor impairment in patients with stroke suggesting reduced levels of motor impairment.

Deng et al. used an 8-camera motion capture system to measure and analyze various gait kinematic parameters to predict fall risk and measure improvement of energy utilization (22). Both groups in their study received ankle movement training via telerehabilitation, however the complexity of the intervention varied. They noted a significant within-group difference with the more complex intervention showing an improvement in ankle dorsiflexion range, toe clearance and stride length. Limb symmetry measured using the gait temporal symmetry ratio improved in both groups.

Bernocchi et al. used a 10-item satisfaction questionnaire at the end of the telerehabilitation program and reported an overall high satisfaction and acceptance of the intervention (19). Lloréns et al. administered the System Usability Scale to measure the usability of their virtual reality-based balance program and the Intrinsic Motivation Inventory which is intended to assess participant's subjective experience related to the intervention in research studies (25). They showed no significant between group differences with high scores in both the in-person and telerehabilitation groups.

Participant adherence, measured by the rate of participant drop out appears to be high with telerehabilitation. Amongst all the included studies, only thirteen out of 248 participants dropped out of the trials, suggesting a good adherence to telerehabilitation.

## Discussion

In this systematic review, we aimed to determine the effectiveness of telerehabilitation on walking function in adult stroke survivors. One of the main findings of our review is the limited number of studies that met our robust inclusion criteria, possibly influenced by the intentional inclusion of bidirectional mode of delivery for the telerehabilitation protocol in order to increase rigor and adherence to training. Seven out of the eight studies included in this review reported improvements in at least one measure of walking function. Seven studies also reported improvements in dynamic balance (19–21, 23–26) and two studies demonstrated improvements in endurance (19, 26). We encountered numerous types of interventions for delivering care via telerehabilitation. Some studies used conventional methods such as sit to stand transition training and overground walking or using virtual reality, while one used neurodevelopmental theory-based treatment and another study focused on ankle movement-based training. Despite this heterogeneity, we saw an overall improvement after telerehabilitation irrespective of intervention type.

### Interventions

Most studies focused on balance training whereas only three studies included walking as a part of the protocol (19, 20, 26). Wu et al. demonstrated superior balance improvements in the telerehabilitation group when compared to the control group (26). This could be because their control group was only provided rehabilitation guidance via telephone and no conventional therapy was administered. Chen et al. (20) included balance and walking training during the acute phase for 12 weeks, but administered 2.5 times the number of sessions conducted by Wu et al. (26). Though they saw a significant improvement within groups, the telerehabilitation group did not show greater improvements in comparison to Wu et al. (26). In the studies that administered virtual reality (21, 24, 25), Chen et al. (21) showed greater balance improvements in the telerehabilitation group compared to the control. This could be because they included virtual target-oriented tasks along with Tai Chi exercises whereas Lin et al. (24) and Lloréns et al. (25) included only static and dynamic balance training. Bernocchi et al. (19) and Huzmeli et al. (23) included more conventional forms of therapy in their intervention group, however both studies did not include a control group altogether, thus between-group comparisons could not be made.

### Walking outcomes

Bernocchi et al. (19) and Wu et al. (26) achieved minimal clinically important differences (MCID) on the 6MWT in acute and subacute stroke respectively, noting similar improvements with telerehabilitation in these two phases of stroke (27). Lloréns et al. showed improved walking ability in both groups on the POMA scale (25). However, these improvements were not clinically meaningful (28). Deng et al., the only study that measured walking speed, did not see an increase in walking speed (22). A potential reason for not observing a difference in gait speed between or within groups could be because their participants received training that focused only on ankle movements and did not include any form of functional gait training. Walking speed is an important indicator of improvement in walking ability and a limiting factor of studies included in this review is the absence of walking speed as an outcome measure.

Improvements in balance were observed in all studies for the telerehabilitation group and two studies reported greater improvements in the telerehabilitation group compared to control. A variety of tests were used to measure balance across studies with BBS being the most common. Four studies (19, 21, 25, 26) assessed more than one outcome measure of balance and three of them showed statistically significant improvements for all balance outcomes (19, 25, 26). Three studies (19, 20, 26) that recruited participants in the acute and subacute stages of stroke, achieved MCID on the BBS (29, 30). Four studies in chronic stroke survivors also used the BBS, however we cannot comment on whether improvements were clinically relevant as the MCID value has not been established in chronic stroke. Bernocchi et al. did not see a clinically relevant change with the Tinetti Performance-Oriented Mobility Assessment (28), possibly because their intervention did not include balance specific training. Some studies showed clinically relevant changes in one balance outcome but not others. For example, Wu et al. (26) showed greater improvements on both the TUG test and BBS scores, while Chen et al. (21) showed improvements on the TUG and not BBS. This could be due to differences in the duration of training and stroke phase. The intervention administered by Chen et al. (21) spanned 4 weeks in chronic stroke whereas, Wu et al. (26) administered 12 weeks of intervention in acute stroke. Despite these differences, TUG test (31) scores were clinically relevant in both studies. Additionally, Lloréns et al. (25) achieved MCID on the BBA (32) but did not show the same clinically important differences on the POMA balance subscale (28). Studies which did not see such clinically relevant improvements in balance did not focus on balance training but focussed on upper and lower limb strengthening exercises.

### Caregiver support

Another factor we considered was the need for caregiver support during delivery of telerehabilitation. All studies necessitated the need for caregiver presence, however only Chen et al. (20) and Huzmeli et al. (23) reported feedback. Chen et al. (20) noted no additional strain on the caregiver in their study while, Huzmeli et al. (23) reported limitations in the caregiver's freedom due to the need for their presence during telerehabilitation. We noted that Chen et al. (20) provided explicit exercise instructions to caregivers and participants, and taught the caregivers how to maintain training logs, while such instructions were not reported by Huzmeli et al. (23). Both interventions included upper and lower limb exercises while Chen et al. additionally administered ETNS along with balance and walking training (20). Surprisingly, Chen et al. (20) also had a longer training duration along with a greater number of sessions with 60 sessions for a duration of 12 weeks while Huzmeli et al. (23) administered 9 sessions over a period of 3 weeks.

### Safety and other factors

Fall risk is one of the most important considerations while designing a telerehabilitation protocol which focuses on walking function. While no objective measures of safety were mentioned in any of the included studies, no study reported adverse events either. This may be attributed to the presence of caregivers throughout the duration of the intervention. It is also possible that adverse events were not encountered as the interventions used in these studies were designed carefully keeping remote administration in consideration. Without any detailed information regarding measures of safety from these studies, it is difficult to provide any further recommendations on safety strategies to be employed in future telerehabilitation protocols.

Another factor to consider for implementation of telerehabilitation is cost-effectiveness. Lloréns et al. was the only study that measured the cost-benefits of telerehabilitation, and concluded that in-person therapy is more expensive than telerehabilitation (25). Expenses were primarily reduced with respect to transportation costs. However, whether the cost savings provide benefits greater or equal to in-person services is unclear at this point.

One of the predicted limitations of telerehabilitation is the dependency on technology literacy at the client's end. Chen et al. noted that users were not required to possess computer skills prior to the intervention as their interface was easy to use and did not affect delivery of therapy (21). On the contrary, Huzmeli et al. reported that participants requested in-person rehabilitation at home instead of the ongoing telerehabilitation sessions (23). They also experienced internet connection issues and electricity disruption. This difference in results could be attributed to the varying geographical locations of the two studies. Accessibility of services should be considered before implementing a telerehabilitation program. Telerehabilitation might not be feasible in certain regions such as rural areas and/or developing countries. There might also be a need for more in-depth participant education before administering telerehabilitation in certain populations.

### Limitations and future recommendations

A major consideration while interpreting results from this review is that we included balance as a part of walking ability. Achieving adequate functional balance is an important goal in rehabilitation for improving walking ability, as seen by strong correlations between balance impairment and ambulatory function (33). An improvement in general balance could contribute to improvements in gait; however, it does not predict better walking in terms of speed or endurance. Caution must be exercised by the reader while interpreting these results with respect to gait. Further studies utilizing walking outcomes such as gait speed and symmetry are needed to warrant a true conclusion regarding the effects of telerehabilitation on walking function.

Another limitation of this review is that we included participants in all phases of stroke due to paucity of literature in this area. It must be kept in mind that acute and subacute stroke participants may still be within their window of recovery, and their results may not be generalizable to those in the chronic phase.

The search criteria for our review was narrow, as we included studies which used bidirectional communication during the telerehabilitation sessions via remote monitoring. We rationalized that supervised therapy would increase the rigor of studies included in this review. This led to exclusion of many articles during our study selection process, which may be a limiting factor for future reviews to consider. Limitations in the validity of the included studies also warrants caution during interpretation of findings. One limitation is the absence of long-term follow-up. We do not know if functional improvements after telerehabilitation were sustained after treatment and if longer protocols or maintenance sessions are needed. Only half of the articles included were rated as good to excellent on the PEDro scale (20, 24–26). Quality of articles were mainly limited by factors such as the inability to blind both the therapists and subjects due to the nature of the intervention contributing to the risk of bias. Another limitation is that we could not find information in the published studies regarding whether interventions were administered by a trained physical therapist. Reporting of stringent training protocols may help consolidate results of future studies.

Lastly, the absence of a control in almost half the studies is a major drawback preventing us from drawing strong conclusions about the superiority of telerehabilitation over conventional therapy. Further studies with a control group receiving the same type of intervention in an in-person setting is needed for a true comparison.

## Conclusions

In summary, conclusive evidence regarding telerehabilitation as an effective mode of delivery of therapy for walking could not be reached as a majority of the included studies did not measure walking speed or endurance. However, positive evidence was found for the use of telerehabilitation for improvements in balance, which has implications for gait recovery. Additionally, the telerehabilitation group showed similar (19, 20, 23–25) and in some cases (21, 26) greater benefits when compared to control and/or in-person therapy in studies that employed a control group. Higher quality studies with more detailed protocols and outcome measures are required to conclude whether telerehabilitation is a superior mode of treatment to conventional therapy for walking function.
